# Genome‐wide association of dry (Tamar) date palm fruit color

**DOI:** 10.1002/tpg2.20373

**Published:** 2023-08-24

**Authors:** Shameem Younuskunju, Yasmin A. Mohamoud, Lisa S. Mathew, Klaus F. X. Mayer, Karsten Suhre, Joel A. Malek

**Affiliations:** ^1^ Genomics Laboratory Weill Cornell Medicine‐Qatar Doha Qatar; ^2^ Department of Genetic Medicine Weill Cornell Medicine‐Qatar Doha Qatar; ^3^ Department of Physiology Weill Cornell Medicine‐Qatar Doha Qatar; ^4^ Clinical Genomics Laboratory Sidra Medicine Doha Qatar; ^5^ School of Life Sciences, Technical University of Munich Munich Germany; ^6^ Plant Genome and Systems Biology Helmholtz Center Munich Munich Germany

## Abstract

Date palm (*Phoenix dactylifera*) fruit (dates) are an economically and culturally significant crop in the Middle East and North Africa. There are hundreds of different commercial cultivars producing dates with distinctive shapes, colors, and sizes. Genetic studies of some date palm traits have been performed, including sex determination, sugar content, and fresh fruit color. In this study, we used genome sequences and image data of 199 dry dates (Tamar) collected from 14 countries to identify genetic loci associated with the color of this fruit stage. Here, we find loci across multiple linkage groups (LG) associated with dry fruit color phenotype. We recover both the previously identified *VIRESCENS* (VIR) genotype associated with fresh fruit yellow or red color and new associations with the lightness and darkness of dry fruit. This study will add resolution to our understanding of date color phenotype, especially at the most commercially important Tamar stage.

## Core Ideas

- Genome‐wide association study approach to identify genetic variants associated with the date palm fruit color.
- We find loci across multiple linkage groups associated with the lightness and darkness of dry fruit color.
- This study will add resolution to our understanding of the fruit color phenotype, especially at the Tamar stage.

## 1 INTRODUCTION

Date palm (*Phoenix dactylifera*) is one of the oldest and most economically important fruit crops in the Middle East and North Africa (Chao & Krueger, 2007; Weiss et al., 2012). While there are thousands of cultivars or varieties, likely, only a few hundred are commercially important (Zaid & Arias‐Jimenez, 1999). These cultivars produce fruit (dates) that vary in shape, color, and size. Fruit development and ripening involve many complex biological processes, with color changes of the fruit being an important factor closely associated with the ripening stage (Abbas & Ibrahim, 1998). Dates have five different development stages: Hababauk, Kimri, Khalal, Rutab, and Tamar (Al‐Mssallem et al., 2013; Siddiq & Greiby, 2013). During the first development stages of Hababauk and Kimri, the fruit skin color is whitish‐green. In the Khalal stage, dates partially ripen and gain maximum size and weight and the color changes from green to yellow or red depending on the cultivar. Dates fully mature in the Rutab stage, and the color begins its change to brown. Tamar is the final stage of ripening, during which fruit water content is reduced to less than 25%, sugar content increases to 70%–80%, and the color turns dark brown. Dates are harvested and sold mainly in three different stages of development: Khalal, Rutab, and Tamar. Khalal and Rutab dates are considered fresh, while Tamar dates are dried fruit (Ahmed et al., 1995). Tamar dates are the most commercially significant and are often preferred because they are easy to store, are high in sugar, low in moisture, and have low tannin levels (Awad, 2007). During the fruit ripening process, amino acids act as building blocks for the synthesis of key intermediates and end products (Seymour et al., 2012), and the enrichment of both free amino acids and color or flavor‐conferring phenylpropanoids was observed in the early ripening stage of dates as in other fruits (Diboun et al., 2015).

Dates are classified as climacteric fruit like other commercial fruit such as apples, bananas, and peach, where ethylene functions as a key regulator in the fruit ripening (Abbas & Ibrahim, 1998; Al‐Qurashi & Awad, 2011; Serrano et al., 2001). Fruit ripening is associated with an increase in respiration rate, burst of ethylene production, oxidation process, sugar accumulation, chlorophyll breakdown, pigment synthesis, and other processes (Osorio et al., 2013; Stepanova & Alonso, 2005). Anthocyanin is a class of secondary metabolite synthesized in higher plants and plays a crucial role in pigmentation (red, pink, purple, blue) in fruit and vegetables (Kong et al., 2003; Kayesh et al., 2013). In anthocyanin biosynthesis, the R2R3 MYB transcription factors act as a regulator (Xie et al., 2020). Previous studies from Hazzouri et al. (Hazzouri et al., 2015; Hazzouri et al., 2019) revealed that a retrotransposon insertion (named Ibn Majid) and a start codon mutation in an R2R3 MYB transcription factor encoded by the ortholog of the oil palm VIRESCENS gene (VIR gene) are likely the key genetic changes leading to the red and yellow color phenotype in dates at the Khalal stage (fresh fruit). Studies have shown that fruit pulp ripening (Khalal‐to‐Rutab‐to‐Tamar), which also involves color transition from yellow to brown (Khalal‐to‐Rutab), showed an increase in endogenous abscisic acid (ABA) concentration (Awad, 2007; Shareef & Al‐Khayri, 2020) and is preceded by an arrest of xylem‐mediated water transport (Elbar et al., 2022).

Studies of the genetic basis of date palm traits have increased recently due to the crop's economic importance and the establishment of the required genetic resources for such studies. Genome‐wide association studies (GWAS) are a powerful method for mapping the association between genetic variation and phenotype (Cantor et al., 2010; Korte & Farlow, 2013). Previous GWAS in date palm identified significant associations for fruit sugar content (Hazzouri et al., 2019; Malek et al., 2020), confirmation of previous findings of the sex determination region on Linkage group 12 (Hazzouri et al., 2019; Mathew et al., 2014), and fresh fruit color (Hazzouri et al., 2019). Challenges in conducting GWAS in date palm exist, including the use of clonal propagation, the rare existence of outbred populations, and geographical population structure. The population structure and cryptic relatedness have the potential to confound the GWAS results and can lead to false discoveries (Chen et al., 2016; Horton et al., 2012; Vilhjálmsson & Nordborg, 2013). Along these lines, multiple studies (Chaluvadi et al., 2014; Flowers et al., 2019; Zehdi‐Azouzi et al., 2015), including our own (Mathew et al., 2015) have confirmed two major subpopulations (broadly Western and Eastern populations) in the date palm. Indeed, our recent study reported that at least three and possibly four novel subpopulations contribute to the current population (Mohamoud et al., 2019), making population structure an important consideration in any date palm GWAS. Different algorithms have been developed to correct for population structure and increase the computational efficiency and statistical power in GWAS (Kang et al., 2008; Wang et al., 2014; Zhang et al., 2018b). Fixed and random model circulating probability unification (FarmCPU) is a statistically powerful and computationally efficient GWAS method to control spurious associations (Liu et al., 2016; Wang & Zhang, 2021). The iterative usage of the fixed effect and random effect models in the FarmCPU method incorporates population structure and kinship matrix as covariates and eliminates the false positive and false negative association results. We, therefore, hoped to implement these methods in the challenge of GWAS in the highly structured date palm population.

In this study, we conducted GWAS using 199 date palm samples to identify the significantly associated genetic loci and possible candidate genes with the color variation of Tamar stage fruit. The samples used in this study are extensively diverse in their country of origin and variety, collected from 14 countries.

**Core Ideas**

- Genome‐wide association study approach to identify genetic variants associated with the date palm fruit color.
- We find loci across multiple linkage groups associated with the lightness and darkness of dry fruit color.
- This study will add resolution to our understanding of the fruit color phenotype, especially at the Tamar stage.

## 2 MATERIALS AND METHODS

### 2.1 Sample collection and genome sequencing

The Qatar dates biobank is a collection of fruit samples from across the date palm growing region, including Qatar, United Arab Emirates (UAE), Iran, Saudi Arabia, Egypt, Pakistan, Libya, Tunisia, United States of America (USA), Morocco, Jordan, Sudan, Oman, and Spain (Stephan et al., 2018). We used 199 date samples from the collection, attempting to include the most important commercial cultivars as well as some lesser‐known varieties (Supporting Information S2). DNA from fruit was extracted, and sequencing libraries were constructed from total DNA (Mathew et al., 2015) and sequenced on the Illumina HiSeq 2500/4000 instruments using 150 bp length reads. All sequencing steps were as described in Thareja et al. (2018).

### 2.2 Phenotypic data

Photographs were taken of 5 to 11 Tamar stage fruits from each cultivar, along with a color‐checker card and a ruler as a size standard (Supporting Information S1: Figure S1). Pictures were taken on a white background with a digital camera. For a more detailed description, please refer to Stephan et al. (2018). Out of 199 samples, only 179 samples were available for phenotypic data and used for the association study. We measured fruit color by recording the intensity of the red (R), green (G), and blue (B) color channels (RGB) in each photograph for each fruit using MIPAR Software (Sosa et al., 2014). The color channel intensity of multiple fruit was calculated separately and then averaged for each cultivar. The average intensity of each color channel (R, G, and B) and the ratio of the color channel, such as R/G, R/B, and G/B were used as phenotype data for the association mapping study.

### 2.3 Genome alignment and SNP calling

Raw Illumina reads of 199 samples were processed to remove adaptor sequences using Trimmomatic software (v0.39). We used the “ILLUMINACLIP:2:30:10 MINLEN:50” settings to keep the read pairs with a minimum length of 50 bp for further processing. Adaptor removed reads were aligned to the PDK50 reference genome (PRJNA40349) using bwa mem software (v0.7.15‐r1140) (Li, 2013) with default parameters. Reads with supplementary (split/short) alignments were marked by the bwa mem‐M option. Each sample alignment was validated with “ValidateSam” (Picard) (http://broadinstitute.github.io/picard) to ensure no errors in output BAM files. Before variant calling, duplicate reads were marked using GATK MarkDuplicates (gatk‐4.1.7.0) (DePristo et al., 2011). The following GATK function, “FixMateInformation” and “SetNmMdAndUqTags,” were used to ensure consistent paired‐read information and fix the NM, MD, and UQ tags in the sorted BAM files. Variant calling was performed using GATK HaplotypeCaller with GVCF mode. Reads with mapping quality less than 20 and duplicate marked reads were excluded from the variant call. Hard filter parameters were applied to filter raw single nucleotide polymorphisms (SNPs) and insertions and deletions (INDELS) using bcftools (v1.10.2) (Li, 2011). The raw SNPs were filtered using “QD < 2.0 || FS > 60.0 || MQ < 40 || SOR > 4.0 || MQRankSum ← 10 || MQRankSum > 10 || ReadPosRankSum ← 6” parameters and INDELS were filtered with “QD < 2.0 || FS > 200.0 || ReadPosRankSum < −20.0” parameters. Thereafter, we utilized QC filter on SNP data. We excluded samples with greater than 70% missingness and SNPs with depth > 11,000 summed across the samples in QC analysis. Genotypes were marked as missing if DP was below 10, the genotype call rate  less than 80%, or a minor allele frequency below 0.01. Finally, multiallelic SNP (‐max‐alleles 2), and SNPs with Hardy–Weinberg Equilibrium *p*‐value less than 1 × 10^−6^ (–hwe 1 × 10^−6^) were filtered out using Vcftools (V0.1.16). The resulting samples and SNPs were used for further analysis.

### 2.4 Linkage disequilibrium analysis

Linkage disequilibrium (LD) was calculated using PopLDdecay software (Zhang et al., 2018a) with ‐MaxDist 200‐MAF 0.05 option. The pairwise correlation coefficient (*R* ^2^) was calculated for all SNPs in 200 kb window with minor allele frequency greater than 5% for the whole genome and 18 linkage groups separately (LG). The decay of LD was calculated by plotting pairwise *R* ^2^ onto genetic distance between the SNP pairs using an approach mentioned in Marroni et al. (2011) and then calculated the distance at which pairwise correlation coefficient (*R* ^2^) is half its maximum value as half decay distance.

### 2.5 Genome‐wide association study

GAPIT (v3) R package implemented FarmCPU (Wang & Zhang, 2021) method was used for the genome‐wide association to color phenotype. We ran LD pruning on QC passing SNP data to improve the computational efficiency of the GWAS analysis. LD pruning was executed with plink software (Purcell et al., 2007) using –indep‐pairwise 500 50 0.99 option. To correct population structure, we used the kinship matrix and the first four principal components (PCA) as covariates. PCA and Kinship matrix was generated by GAPIT using LD pruned SNPs data set. VanRaden algorithm in GAPIT R package used for calculating kinship. This study used a false‐discovery rate (FDR) adjusted *p*‐value of 0.05 (5%) as a cut‐off value for identifying the list of significant SNPs associated with date palm fruit color phenotype. QQ‐plot and Manhattan plots were generated from the GWAS result using the R package CMPlot (Yin et al., 2021).

### 2.6 VIR genotyping and significant SNP effects on Tamar stage fruit color

The genomic region of the VIR gene and Ibn Majid retrotransposon from the Barhee BC4 genome (Hazzouri et al., 2019) were aligned against the PDK50 reference genome, and the coordinates of its insertion and termination codon in the VIR gene noted. Following the same procedure used in Hazzouri *et al.* study, we manually inspected all the QC filtered samples to identify the VIR genotypes (VIR^+^/VIR^+^, VIR^+^/VIR^IM^, and VIR^IM^/VIR^IM^) using JBrowse2 software (Diesh et al., 2023). In this study, samples were classified into two groups: (i) homozygous VIR^+^/VIR^+^ genotype; (ii) homozygous VIR^IM^/VIR^IM^ genotype. That is, only homozygous genotypes were used for further analysis to avoid confusion about the effect of the heterozygous genotypes. To investigate the effect of loci on dry fruit color identified in this study, our SNPs were analyzed within each homozygous VIR sample group.

### 2.7 Candidate gene and variant analysis

Regions 150 kb upstream and downstream of the significant SNP markers (total 300 kb) were considered for possible candidate genes and variant analysis. Possible gene sequences and coordinates were mapped from the region using the GFF3 gene annotation file (Supporting Information S3) of the PDK50 reference genome. Gene ontology of mapped gene sequences was performed using Blast2Go software (Conesa & Götz, 2008). Literature search and Blast2Go results were used to identify the function of the candidate genes. All SNPs and INDELs from the potential regions were annotated using SnpEff software (Cingolani et al., 2012). LD *R* ^2^ value of each SNP from the candidate region was calculated against the significant SNPs from the GWAS result using PLINK software. Amino acid substitution effects on the protein function of the candidate SNPs were analyzed using SIFT (Sim et al., 2012).

### 2.8 Structural variation analysis

Clipped, discordant, unmapped, and indel reads from each sample were used for structural variation analysis. The reads were collected from samples having GWAS significant SNPs with homozygous allele alternative (ALT) to the reference genome. Reads from each sample were grouped based on homozygous ALT alleles and assembled separately using SPADES (SPAdes‐3.15.2) (Bankevich et al., 2012) according to the recommended parameters. Assembled contigs were aligned against the reference genome using nucmer (Marçais et al., 2018). We then mapped structural variation breakpoints and annotated them using the Assemblytics software (Nattestad & Schatz, 2016).

### 2.9 RNA seq analysis

RNA‐seq expression analysis was performed using data from Hazzouri et al.’s study (Hazzouri et al., 2019). We used RNA‐seq data of three or four replicates of varying days post‐pollination (45, 75, 105, 120, 135 days) in two fruit varieties, Kheneizi (a variety with VIR^+^/VIR^+^ genotype and produce red fresh fruit) and Khalas (a variety with VIR^IM^/VIR^IM^ genotype and produce yellow fresh fruit). RNA‐seq fastq files were downloaded from SRA (PRJNA505138). The reads were aligned to the reference genome using STAR split read aligner (v2.7.1a) (Dobin et al., 2013) with two pass alignment. Thereafter, the read counts per gene were calculated using the featureCounts program with the following parameter “‐p –countReadPairs ‐t exon ‐g gene_id ‐s 0.” DESeq2 (Love et al., 2014) with the median‐of‐ratio method was used for read normalization.

## 3 RESULTS

### 3.1 Phenotypic data

**FIGURE 1:**
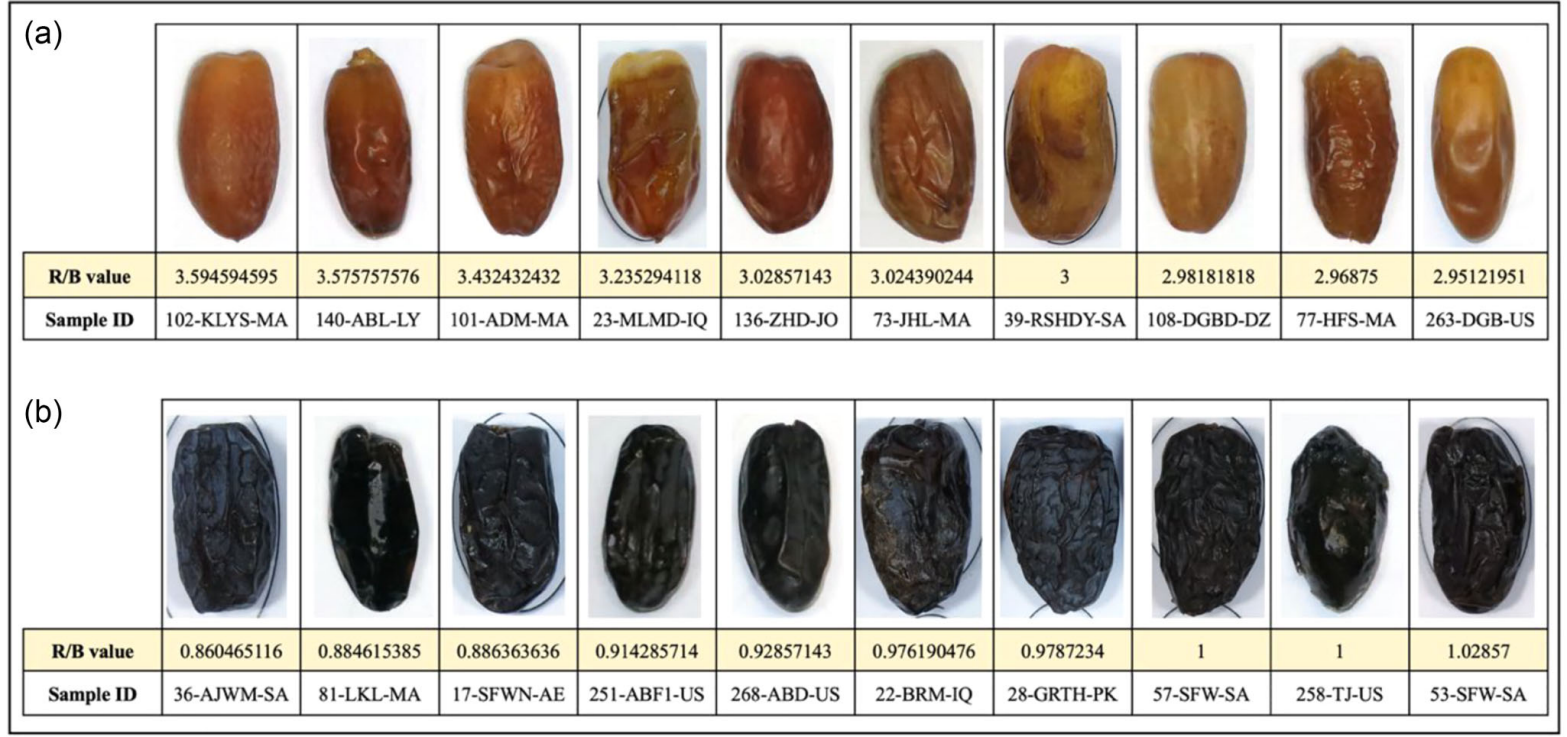
Comparison of fruit images by red/blue channel (R/B) phenotype values. Fruit color ranges from dark to light brown as the R/B value increases (min 0.86, max 3.59). (a) Images of 10 date samples which have the highest R/B values; (b) images of 10 date samples which have the lowest R/B values.

In the Tamar stage, among other changes, date palm fruit color varies from light brown to dark brown. We measured the light intensities of red, green, and blue color channels and ratios of red/blue (R/B), red/green (R/G), and green/blue (G/B) of dry fruit color as phenotypic traits to assess the color variation. Manual analysis of fruit images with the ratio of red and blue channel intensity (R/B) showed that higher values of R/B are associated with light brown and lower R/B values with dark brown (Figure 1). We, therefore, focused our analysis on R/B color phenotype for the genome wide association study of color.

### 3.2 Genome‐wide SNP calling and LD analysis

Genotyping of date palm samples using GATK HaplotypeCaller produced a filtered call set of 31,373,292 SNPs and 8,278,578 indels. Following the application of quality control filters (QC filters), we identified 10,183,993 SNPs across 188 samples, approximately, 12 SNPs/kb. Among those, 9,409,021 SNPs span over 18 linkage groups (LGs), and 774,972 SNPs are in unplaced scaffolds of the pdk50 reference genome.

**FIGURE 2:**
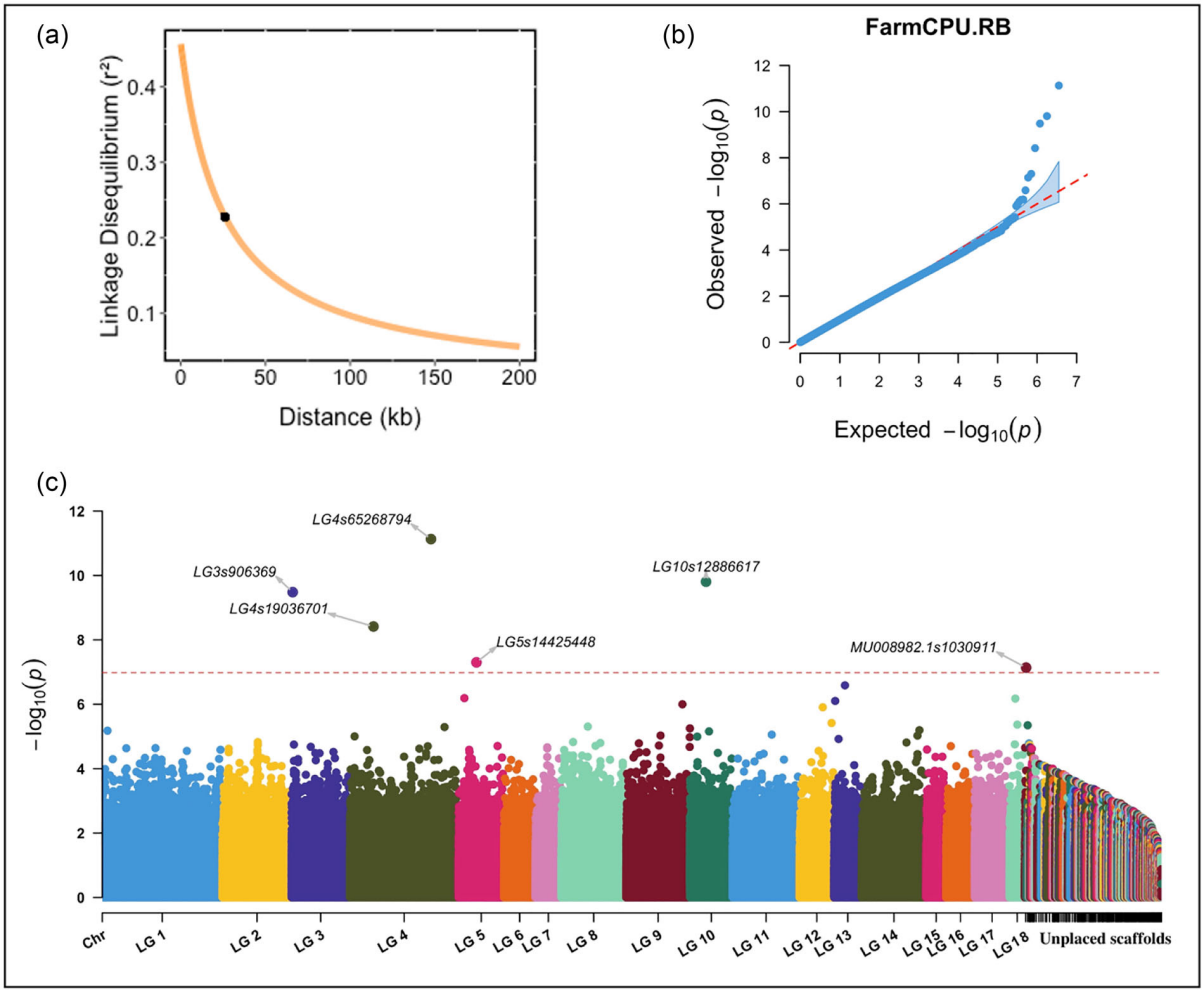
Genome‐wide association study (GWAS) analysis of Tamar stage date fruit color (R/B). (a) Decay of linkage disequilibrium with physical distance in the GWAS mapping population. The half decay distance is 25.9 kb. (b,c) QQ plot (b) and Manhattan plot (c) using the linkage disequilibrium (LD) pruned SNP set (3,541,727 SNPs) for all linkage groups (LG) and unplaced scaffolds. [Correction added on 5 September 2023 after first online publication: Corrections made to image tags as ‘(a), (b) & (c)’ instead of ‘(a), (b), & (b)’.]

Linkage disequilibrium patterns of the genome of GWAS mapping population (*n* = 188), linkage group wise (LG), and subpopulation (western and eastern) were investigated. The pairwise correlation coefficient (*R* ^2^) result of the genomes of the GWAS population showed that the *R* ^2^ value decreased to half of its maximum value (half LD decay) at 25.9 kb (Figure 2a), which is close to the LD decay distance (22.9 kb) found by Hazzouri et al. (2019). Furthermore, the LD decay rate varied among linkage groups (Table 1; Supporting Information S1: Figure S2). LD decay was slower in LG12, and the *R* ^2^ value decreased to half of its maximum value at 38.4 kb, possibly related to this linkage group associated with the sex determination (Mathew et al., 2014). The LD decay was faster in LG 4 and LG 15, with *R* ^2^ value half of its maximum value at 18.9 and 17.2 kb, respectively. Our GWAS mapping population contains 68 samples of the eastern cultivar (36%) and 106 samples of the western cultivar (56.8%). The subpopulation analysis revealed that the *R* ^2^ value dropped to half of its maximum value at 21.73 kb in the eastern cultivar and 35.85 kb in the western cultivar (Supporting Information S1: Figure S3).

**TABLE 1:** Decay of linkage disequilibrium (LD) with physical distance in each linkage groups (LG).

LG ID	Half LD decay (*r* ^2^)	Half LD decay distance (kb)
LG1	0.2282535	27.94
LG2	0.2282521	23.81
LG3	0.2282519	23.53
LG4	0.2282495	18.90
LG5	0.2282531	26.51
LG6	0.2282532	26.92
LG7	0.2282505	20.58
LG8	0.2282518	23.14
LG9	0.2282527	25.48
LG10	0.2282519	23.36
LG11	0.2282527	25.47
LG12	0.2282558	38.49
LG13	0.2282509	21.26
LG14	0.2282532	27.05
LG15	0.2282484	17.27
LG16	0.2282545	31.90
LG17	0.2282533	27.16
LG18	0.2282515	22.44

### 3.3 Association of Tamar stage fruit color to genotype

Association of color channels for Tamar stage fruit (red, green, blue, R/B, R/G, and G/B) using the LD pruned SNPs set (SNPs = 3,541,727) resulted in the identification of several significant (FDR < 0.05) SNPs associated with all colors except blue. We chose to focus on the R/B phenotype as this correlated best to how light or dark a fruit was, as mentioned above (Figure 1). A Q‐Q plot of genome‐wide association results with the R/B phenotype shows a sharp deviation from the expected P‐value distribution in the tail area and a lambda score of 0.98 (Figure 2b), showing good control of both false positives and false negatives. We identified six SNPs that are significantly associated with the dry fruit color phenotype (Table 2 and Figure 2c). These SNPs were located on multiple linkage groups, including LG 3, LG 4, LG 5, LG 10, and one unplaced scaffold MU008982.1. Some of these SNPs overlap with GWAS results of other color channels. A SNP from LG 4 (LG4s65268794) has a highly significant *p*‐value (10 × 10^−12^), and it is common for both the R/B and R/G phenotypes (Figure 2c; Supporting Information S1: Figure S7), while SNP LG10s12886617 from LG10 is common in R/B and G/B phenotypes (Supporting Information S1: Figure S8). The R/B associated SNP regions were selected for further study of possible candidate genes functionally involved in the color phenotype.

**TABLE 2:** List of significant SNPs associated with R/B phenotype from genome‐wide association study (GWAS) result. A false‐discovery rate (FDR)‐adjusted *p*‐value of 0.05 was used as a cut‐off value for identifying the list of significant SNPs associated with the phenotype.

SNP ID	LG ID	SNP position	*p* Value	FDR *p* value	MAF
LG4s65268794	LG4	65268794	7.40 × 10^−12^	2.62 × 10^−5^	0.41620112
LG10s12886617	LG10	12886617	1.56 × 10^−10^	0.000276	0.19273743
LG3s906369	LG3	906369	3.29 × 10^−10^	0.000388	0.29329609
LG4s19036701	LG4	19036701	3.84 × 10^−9^	0.003401	0.17877095
LG5s14425448	LG5	14425448	4.98 × 10^−8^	0.035277	0.08938547
MU008982.1s1030911	MU008982.1	1030911	7.25 × 10^−8^	0.042803	0.20391061

### 3.4 Significant SNP's genotypic effects on the Tamar stage fruit color

**FIGURE 3:**
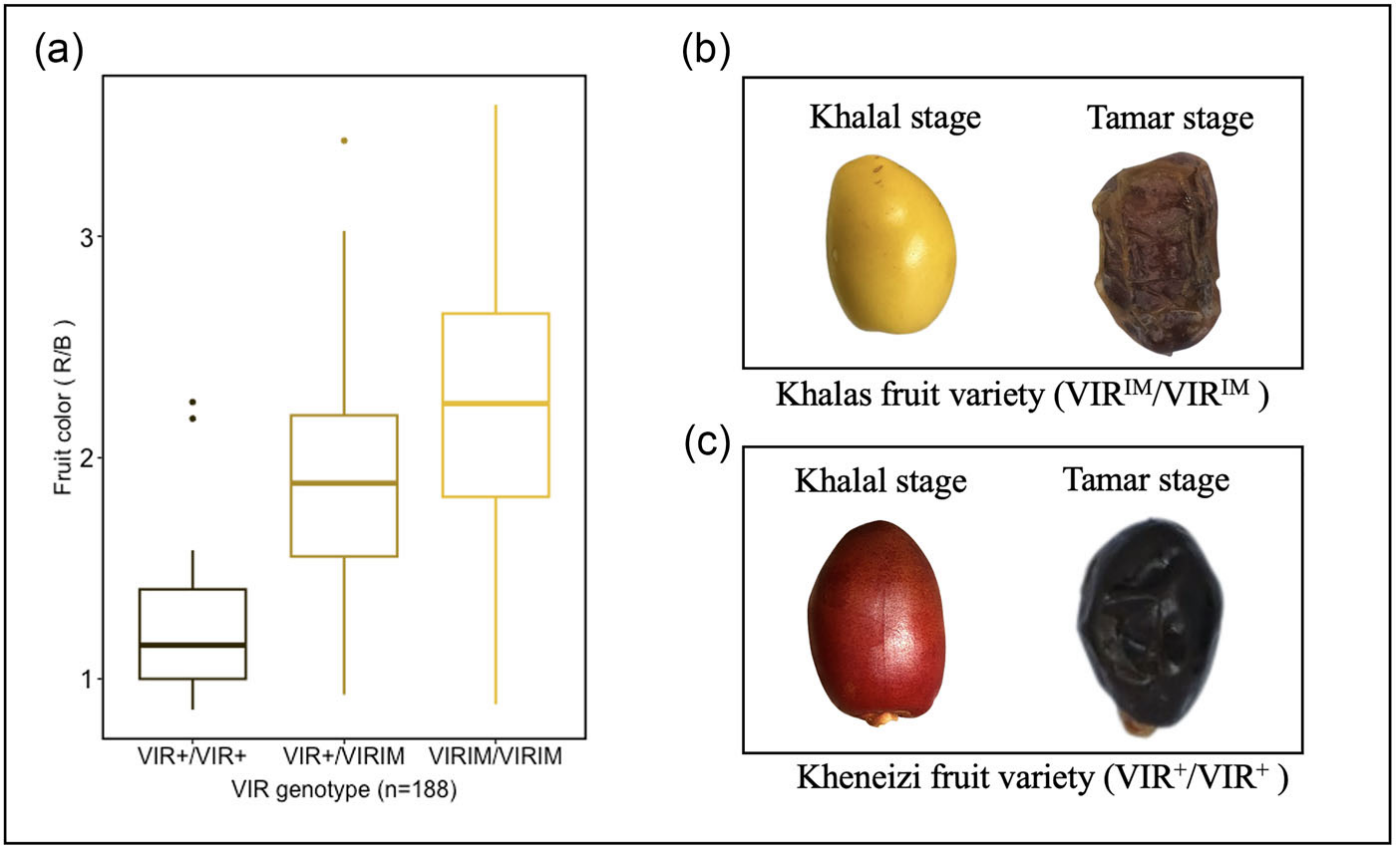
Fruit color phenotype (R/B) analysis by VIR genotype in the current study. (a) Boxplot distributions analysis showing the effect of the VIRESCENS (VIR) genotype on Tamar stage color (R/B). (b,c) Tamar and Khalal stage of Khalas and Kheneizi fruit variety. Khalas has VIRIM/VIRIM genotype, which yields yellow fruit in the Khalal stage and turns light brown in the Tamar stage. Kheneizi has the VIR+/VIR+ genotype (wild type), yielding red fruit in the Khalal stage and dark brown in the Tamar stage. [Correction added on 5 September 2023 after first online publication: The previous image contained stock images for the red and yellow color fruits (khalal stage) and the images have been replaced with the authors' photos.]

Visual inspection shows that allelic variation in the identified SNPs (Table 2) are indeed associated with light (high R/B value) or dark brown color fruit (low R/B value) (Supporting Information S1: Figure S9). Encouragingly, the R2R3 transcription factor gene (ortholog of the oil palm VIR gene), previously reported to regulate red or yellow color at Khalal stage (fresh fruit) (Hazzouri et al., 2015; Hazzouri et al., 2019), is present 16 kb away from SNP LG4s65268794. As validation, we separated our samples by the VIR genotype and compared them to their color. Our results show that fruits were classified into light brown, dark brown, and mixed colors of light and dark fruit based on the VIR genotype (Figure 3a). Out of 188 samples, 27 samples are homozygous wild type (VIR^+^/VIR^+^) and are dark brown, while 61 are homozygous for transposon insertion (VIR^IM^/VIR^IM^) and are light brown; 100 of our samples are VIR^+^/VIR^IM^ genotypes and, accordingly, show a mixed range of color (light & dark brown) in which VIRIM acts as dominant or semi‐dominant by interfering with the expression of the wild‐type VIR^+^ allele. We also identified the start codon mutation (ATG to ATA) of the R2R3 transcription factor gene (called as VIR^saf^) in homozygous wild‐type samples, previously reported in Hazzouri et al.’s study (2019). VIR^+^ is haplosufficient in VIR^+^/VIR^saf^ genotype and produces dark brown fruit like homozygous wild type (Hazzouri et al., 2019).

**FIGURE 4:**
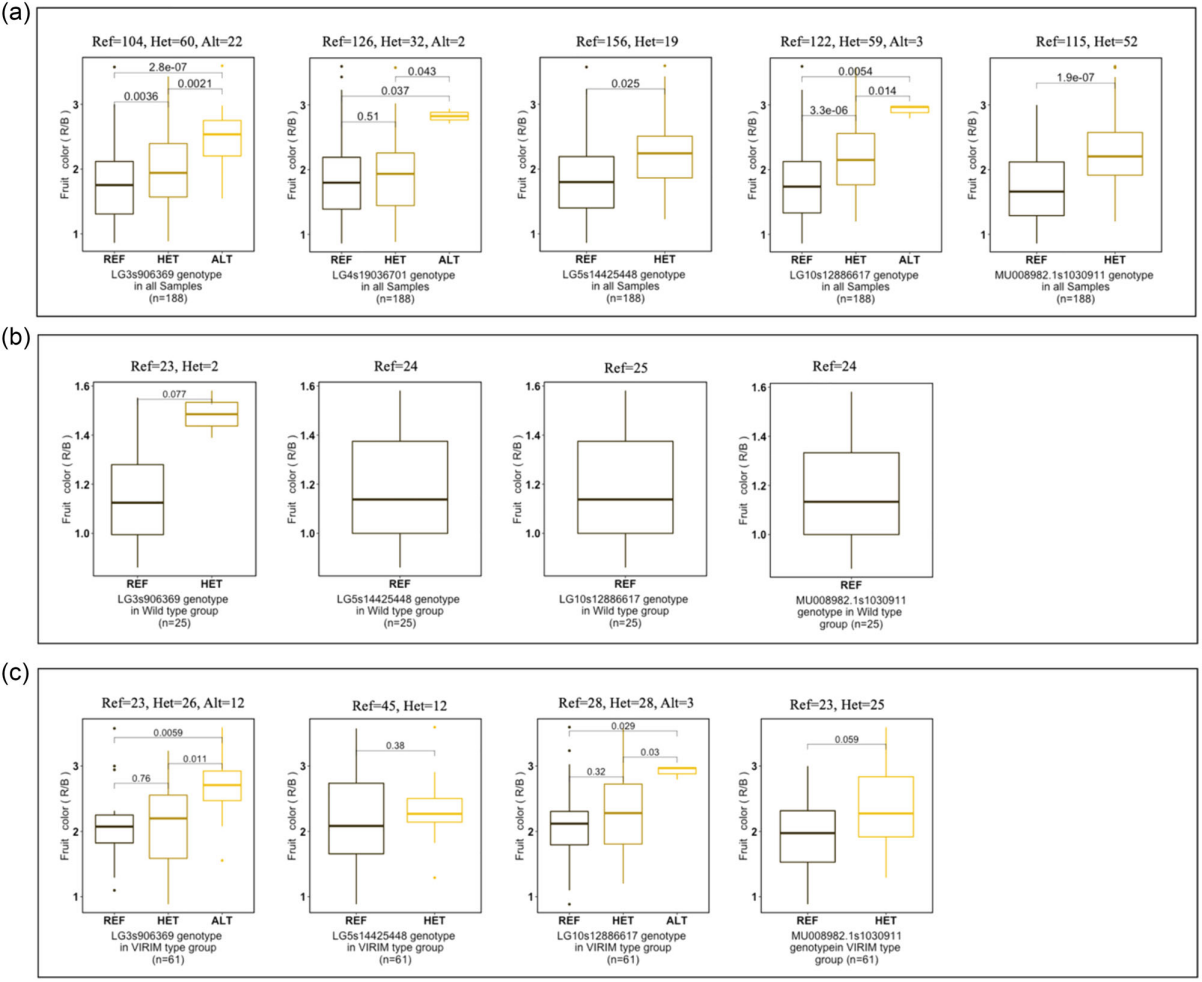
Boxplot distribution analysis of color phenotype (R/B) by genotypes of significant Genome‐wide association study (GWAS) SNPs from this study. Samples were classified into three groups: (a) all 188 samples group, (b) wild type VIR group: samples with VIR+/VIR+ or VIR+/VIRsaf (*n* = 25), and (c) VIRIM group: samples with VIRIM/VIRIM genotype (*n* = 61) prior to analysis of phenotype by genotypes of significant SNPs identified in this study. *p*‐Values were calculated using Wilcoxon statistical test. The *X*‐axis represents the SNPs’ genotypes, and *Y*‐axis represents R/B phenotypic value. Results show significant association with fruit color even when samples are separated by the VIR genotypes associated with red/yellow fresh fruit color. Genotypes of SNP LG4s19036701 could not distinguish the color in the VIRIM sample group, likely because of linkage on the same chromosome as the VIR gene, and so was excluded from this specific analysis (Supporting Information S1: Figure S10).

**FIGURE 5:**
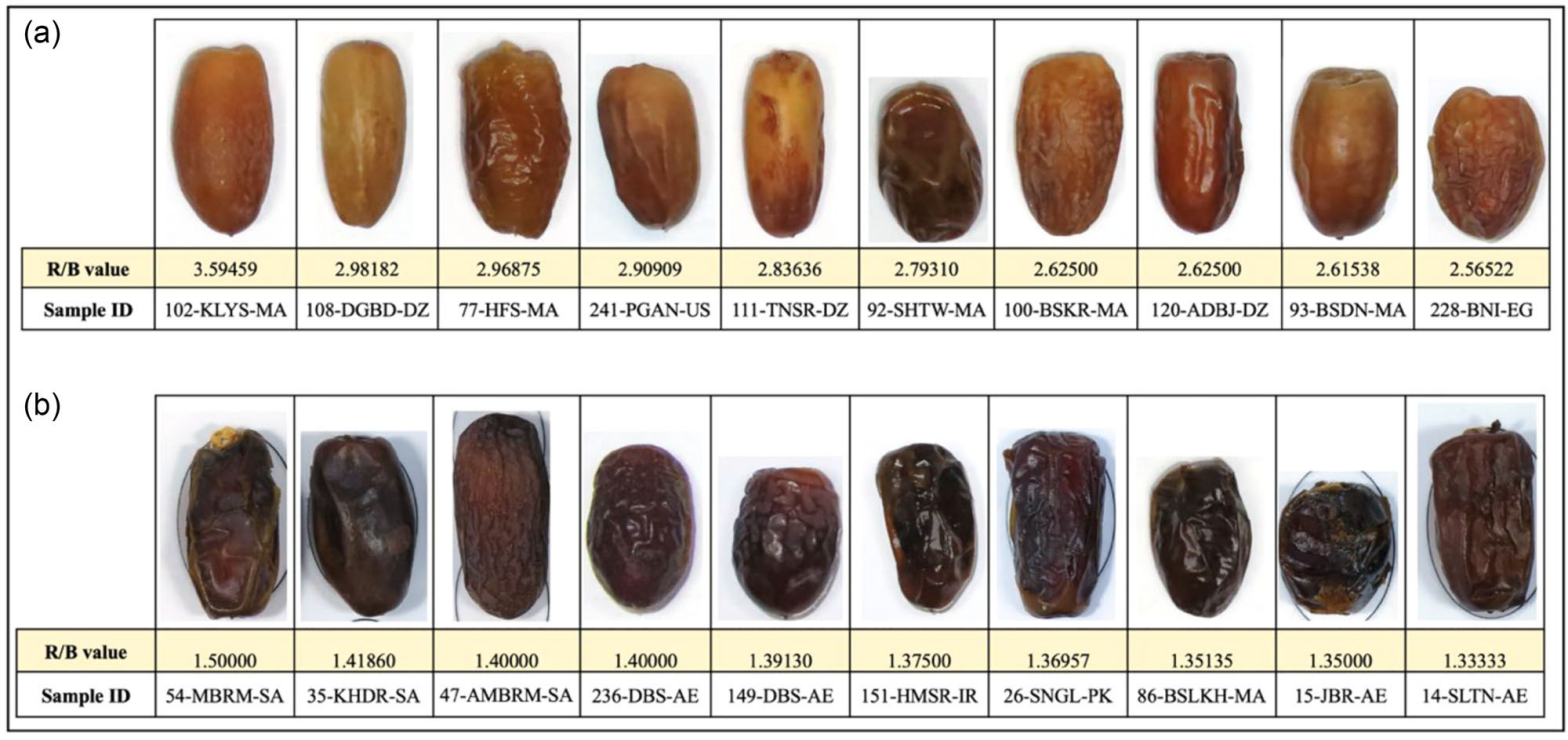
Fruit color differences within the homozygous VIRIM sample group (yellow fresh fruit color) when categorized by the genotypes of SNP LG3s906369. Samples were grouped as homozygous for the (a) ALT or (b) REF allele of SNP LG3s906369. Results show that the lightness of the color increases (R/B value increases) when the sample is homozygous ALT allele at SNP LG3s906369.

For further understanding of the color variation in dry fruit beyond the R2R3 transcription factor, we visualized fruit color based on the genotype of GWAS identified SNPs (Table 2). The analysis was conducted in all 188 samples and then in groups separated by VIR genotype (Figure 4). The analysis results of the combined 188 samples showed significant associations (Wilcoxon test) with the color phenotype as expected (Figure 4a) given the major role the VIR genotype plays in color. However, analysis of sample groups first categorized by their VIR genotype also showed significant association between some SNPs and dry fruit color. That is, even within the various VIR genotype groups that are key to red and yellow fresh fruit color, the SNPs identified here often further distinguish Tamar stage fruit color significantly (Figure 4b,c), suggesting these regions may play a role in dry fruit color or a more fine‐grain role in fresh fruit color that was previously undetected. The result from the homozygous VIR wild‐type sample group, excluding the VIR^saf^/VIR^saf^ genotype samples (total of 25 samples), shows that fruit's darkness decreases (R/B value increases) when the sample is heterozygous for SNP LG3s906369 while darkness increases (R/B value decreases) when the sample is homozygous for the reference allele (Figure 4b). SNPs LG5s14425448, LG10s12886617 and MU008982.1s1030911 have only homozygous REF alleles in the group, so were not considered for this analysis. The homozygous VIRIM sample group shows that the lightness of the fruit color increases (R/B value increases) when the sample is homozygous ALT or HET allele of SNP LG3s906369, LG5s14425448, LG10s12886617, and MU008982.1s1030911 (Figure 4c). Altogether, these results show that the SNPs identified here are associated with dry fruit color beyond the previously identified VIR gene associated with fresh fruit color. Manual inspection of fruit color separated by the genotypes of SNP LG3s906369 within the homozygous VIRIM group supports this (Figure 5). Sample group and genotype information are detailed in Supporting Information S4. [Correction added on 5 September 2023 after first online publication: The citaion “Figure 3b” is changed to “Figure 4b”.]

### 3.5 Candidate gene and SNP annotation

We considered potential candidate genes when they were within 150 kb upstream to 150 kb downstream of the significantly associated SNP. While this is broad given average LD decay, we wanted to ensure the possibility of capturing linked genes that may be outside the standard LD decay size. There were 120 genes present across all the potential regions of significant SNPs from the GWAS result of the R/B color phenotype (Supporting Information S5). Putative gene functions from blast2Go and literature searching show that many genes related to fruit ripening and pigmentation are present within these regions (Table 3), and many are present within 50 kb of the significant SNPs.

**TABLE 3:** List of genes detected around the 300 kb region of significant SNPs from genome‐wide association study (GWAS) result associated with the R/B color phenotype. Genes were selected if they have a putatively significant role in fruit ripening and pigmentation.

Tag SNP	LG ID	Gene description	Distance from Tag SNP (gene location)
LG4s65268794	LG4	R2R3‐MYB transcription factor	16 kb
LG10s12886617	LG10	Ethylene‐responsive transcription factor12	116 kb
LG10s12886617	LG10	RING/U‐box superfamily protein	3 kb
LG10s12886617	LG10	Myb family transcription factor family protein	12 kb
LG3s906369	LG3	Protochlorophyllide reductase, chloroplastic	3.5 kb
LG3s906369	LG3	Basic helix‐loop‐helix (BHLH) DNA‐binding superfamily	83 kb

**FIGURE 6:**
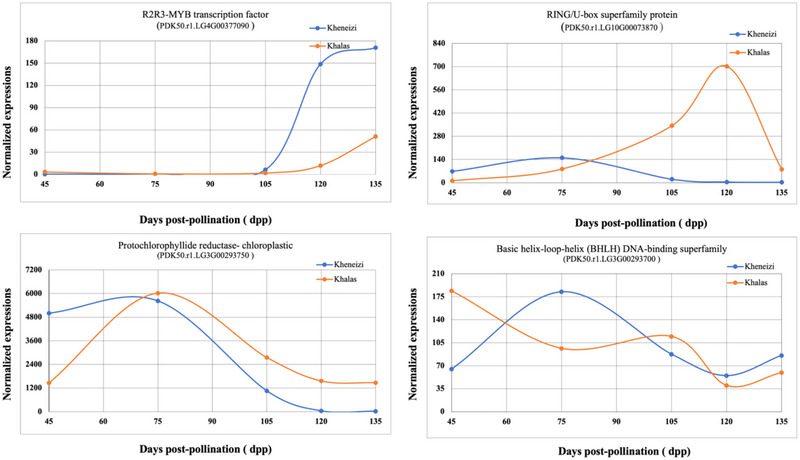
RNA seq analysis of expression of genes related to fruit ripening and pigmentation are present within the region around identified significant SNPs. The gene expression analysis was performed across three or four replicates of fruit development stages of two varieties, Kheneizi (red) and Khalas (yellow). Each point of the *X*‐axis is the development stage (post‐pollination date) and the *Y*‐axis is the mean of normalized expression read count across three or more replicates.

Gene expression analysis using the RNA‐seq data for Khalas (yellow fresh fruit) and Kheneizi (red fresh fruit) varieties shows that many genes from the potential candidate regions were expressed during the various days post‐pollination (dpp) (Figure 6). RNA seq data of different fruit development stages, including Kimri (dpp 45–75), Khalal (dpp −105), and Rutab (dpp 120–135) of both varieties from the Hazzouri et al. study were included (see Section 2). In date palm, the fruit ripening starts from the Khalal stage (fresh), and the color turns red/yellow from green depending on the variety and then turns brown in the Rutab stage (matured). The R2R3 transcription factor gene from LG 4 was expressed during the late stages of development (Khalal to Rutab) in both cultivars (dpp 105, 120, and 135). However, the expression of the R2R3 transcription factor gene was lower in the yellow variety compared to the red ones. RING/U‐box superfamily protein from LG 10 was expressed at dpp 105 to 120 (Khalal to early Rutab stage) in the yellow variety and peaked at dpp 120 compared to the red one. Other genes, such as basic helix‐loop‐helix (BHLH) DNA‐binding superfamily (LG 3), protochlorophyllide reductase (LG 3), was expressed early in the development stage (Kimri) and reduced expression late in the development stage (Khalal to Rutab stage) in both fruit varieties.

We conducted structural variation analysis on all potential regions from all LGs. The analysis showed a 5 kb deletion in the candidate region of SNP LG10s12886617 on LG 10 (Supporting Information S1: Figure S11). While this 5 kb deletion is located 800 bases away from the pentatricopeptide repeat‐containing protein gene and 7 kb away from the SNP LG10s12886617, the association of its presence/absence to the GWAS SNP was not high enough to warrant further investigation. The SnpEff annotated SNPs and INDELs from the potential candidate genes with LD *R* ^2^ value > = 0.6 to the GWAS‐identified SNPs revealed numerous putative high and moderate effect variations to encoded proteins (Supporting Information S6). Among this is one of the SNP LG10s12771512 that is within the ethylene‐responsive transcription factor12 gene (Table 3), and SIFT (Sim et al., 2012) results of amino acid substitution effects on protein function show that it is putatively deleterious.

## 4 DISCUSSION

Commercial dates are most often consumed at the Tamar stage (dry fruit) (Siddiq & Greiby, 2013). Fruit harvested at this stage has long shelf life and can be used for year‐round consumption. The commercial grade of dates is based on phenotypes such as color, size, skin texture, sweetness, and flavor (Chao & Krueger, 2007). Understanding the genetic markers and genes associated with these phenotypes can provide valuable insights for enhancing fruit quality and potentially developing new cultivars with desirable traits (Mir et al., 2019; Yang et al., 2014). We identified six significant SNPs associated with color of dry fruit, significantly extending knowledge beyond previously identified markers for fresh fruit color. We confirmed that the previously identified VIR genotype has a significant effect on dry fruit color, likely stemming from the starting color at the fresh fruit stage of yellow or red. However, beyond this, our results revealed that the newly identified SNPs could give further resolution to the lightness of the brown color of dry fruit even when samples were separated by VIR genotype (Figure 4b,c). For some SNPs, we think that low numbers of samples in the two groups (wild type group = 25 and VIRIM group = 61) might be the reason for the lack of the statistically significant association. However, the overall picture is that the newly identified loci are associated with the color phenotype and can distinguish the dry fruit color beyond simply the color observed in fresh fruit. This association may relate to genetic control during the fruit ripening process.

The pigmentation changes during fruit ripening is a complex process, and many genes related to anthocyanin biosynthesis, flavonoids, and phenylpropanoids are involved in the process (Falchi et al., 2013; Fiol et al., 2022; Ma et al., 2014; Wang et al., 2020). In our study, six genes identified in the regions surrounding the significant SNPs (Table 2) are predicted to play a role in fruit ripening and pigmentation in other plants (Table 3). RNA‐Seq analysis revealed that many of these genes are differentially expressed at various times in the development stages of two different color varieties of fruit (Figures 3b,c and 6). The ethylene‐responsive transcription factor‐12 gene, present in the candidate genomic region of SNP LG10s12886617 (LG 10), is similar to the DORNROSCHEN‐like protein in *Arabidopsis thaliana*. It contains the AP2 domain and has a significant role in the signaling pathway of cytokinin and ethylene activation (Das et al., 2012; Phukan et al., 2017). In *Arabidopsis*, cytokinin signaling increases the sugar‐induced anthocyanin biosynthesis (Das et al., 2012). The candidate region from LG10 also contains an uncharacterized protein (gene id: PDK50.r1.LG10G00073880) which contains Myb DNA‐binding 3 domain (Ambawat et al., 2013). The protochlorophyllide reductase gene is present within the region surrounding LG3s906369 SNP. This gene plays a vital role in chlorophylls' biosynthetic pathway (Garrone et al., 2015; Yamazaki et al., 2006).

SNPs that were filtered out due to high FDR yet remained near the top of our list also identified regions with many genes related to fruit ripening and pigmentation. These sub‐FDR cutoff regions (Supporting Information S1: Table S2) include the candidate region of SNP LG13s8766984 (LG 13), which contains an AP2‐like ethylene‐responsive transcription factor. Other genes include 4‐coumarate‐CoA ligase and 4‐coumarate:coA ligase 3, Myb family transcription factor family protein, AP2/B3‐like transcriptional factor family protein, and chalcone‐flavanone isomerase that are present around the region of SNP LG5s4683788 (LG 5). Chalcone‐flavanone is a critical enzyme for the anthocyanin biosynthesis (Kang et al., 2014; Sun et al., 2019). The 4‐coumarate: CoA ligase is a key enzyme in phenylpropanoid metabolism in plants (Li et al., 2015; Wang et al., 2016), and previous metabolome analysis detected enrichment of phenylpropanoids in the early development of dates (Diboun et al., 2015). Our gene expression analysis shows 4‐coumarate: CoA ligase genes (gene id: PDK50.r1.LG5G00393990 and PDK50.r1.LG5G00393890) are highly expressed early in red color fruit compared with yellow color, peaking at 45–75 days post pollination (Supporting Information S1: Figure S13). These genes may provide candidates for further study if larger sample numbers reveal them to be indeed significantly associated with fruit color.

Many commercially important fruits such as apricots, prunes, figs, cranberry, grape (raisin), and dates are frequently consumed in the dry stage and many have been genetically characterized for fresh fruit color phenotypes (Diaz‐Garcia et al., 2018a; Diaz‐Garcia et al., 2018b; Gundogdu et al., 2017; Hazzouri et al., 2019; Lijavetzky et al., 2006). These studies revealed that in addition to environmental factors, fruit color is strongly associated with genetic variation in or expression of genes related to anthocyanin biosynthesis and phenolic compounds. The accumulation of phenolic compounds has been found to impact the lightness and darkness of the fruit color (Piljac‐Žegarac et al., 2009). Studies on apricot and fig have shown that a higher concentration of phenolic compounds result in dark‐skin‐colored fruit compared to light‐skin‐colored fruit (Ercisli et al., 2012; García‐Gómez et al., 2022; Gundogdu et al., 2017). Despite all the studies of these fruit at the fresh stage, we are not aware of any for dry fruit color, specifically. We believe that our association study is the first study to identify markers associated specifically with dry fruit color and that within the context of a strong genetic factor determining fresh fruit color. In this study, by combining the genotypic data of diverse samples collected from 14 countries and the color phenotype, we successfully conducted a GWAS and identified multiple significant SNPs associated with the color variation of dry dates. Candidate genes involved in the color changes were identified in the associated region and offer the opportunity for further functional studies. The finding from this study helps add resolution to our understanding of genetic control of commercially important phenotypes in dates and potentially other dried fruit crops.

## AUTHOR CONTRIBUTIONS

**Shameem Younuskunju**: Data curation; Formal analysis; Investigation; Methodology; Software; Validation; Visualization; Writing‐original draft. **Yasmin A. Mohamoud**: Data curation; Methodology; Project administration; Resources. **Lisa S. Mathew**: Data curation; Methodology. **Klaus F. X. Mayer**: Conceptualization; Supervision; Writing‐review & editing. **Karsten Suhre**: Conceptualization; Funding acquisition; Supervision; Writing‐review & editing. **Joel A. Malek**: Conceptualization; Funding acquisition; Investigation; Resources; Supervision; Validation; Writing‐review & editing.

## CONFLICT OF INTEREST STATEMENT

On behalf of all authors, the corresponding author states that there is no conflict of interest.

## Supporting information

Supporting Information S1

Supporting Information S2

Supporting Information S3

Supporting Information S4

Supporting Information S5
