# Supplementary material for: Genome‐wide association of dry (Tamar) date palm fruit color
Source: Plant Genome. 2023 Aug 24;16(4):e20373. doi: 10.1002/tpg2.20373 (PMC12806862; doi:10.1002/tpg2.20373)
Supplement: Supplementary file 1 [file TPG2-16-e20373-s003.docx]

Supplemental Material

**Supplementary Figures**

Supplementary Figure 1. Fruit photographs are used for measuring the color intensity of fruit color phenotype. The average intensity of each color channel (Red, Green & Blue) from each fruit image was calculated and used as phenotypic data for the association mapping study.

Supplementary Figure 2. Decay of Linkage Disequilibrium (LD) with physical distance in each Linkage group (LG). Points on the decay curve represent the half decay distance for each LG.

Supplementary Figure 3: Decay of Linkage Disequilibrium (LD) with physical distance in Western and Eastern cultivars. The GWAS mapping population (n=188) contains 68 eastern and 106 western samples. The decay of LD with physical distance was calculated in (a) western and (b) eastern populations separate. Points on the decay curve represent the half decay distance at 35.85 kb and 21.73 kb for western and eastern populations.

Supplementary Figure 4. Association of Red color channel phenotype of Date palm. Manhattan plot and QQ plot using the LD pruned SNPs set (3,541,727 SNPs).

Supplementary Figure 5. Association of Green color channel phenotype of Date palm. Manhattan plot and QQ plot using the LD pruned SNPs set (3,541,727 SNPs).

Supplementary Figure 6. Association of Blue color channel phenotype of Date palm. Manhattan plot and QQ plot using the LD pruned SNPs set (3,541,727 SNPs).

Supplementary Figure 7. Association of R/G color channel phenotype of Date palm. Manhattan plot and QQ plot using the LD pruned SNPs set (3,541,727 SNPs).

Supplementary Figure 8. Association of G/B color channel phenotype of Date palm The association study used the LD pruned SNPs set (3,541,727 SNPs).

Supplementary Figure 9. Boxplot distribution analysis of fruit color phenotype by genotypes of GWAS significant SNPs associated with fruit color (R/B) in all 188 samples. The significant SNPs were identified using FDR adjusted p-value of 0.05 as a cut-off value.

Supplementary Figure 10. Genome mis-assembly correction of R2R3 Myb transcription factor gene sequence in PDK50 genome. PacBio sequencing errors resulted in three indel mutations occurring at exon positions 330, 346, and 352 of the gene. Unfortunately, these errors could not be resolved during pilon genome polishing due to low read co	verage (minimum of 10 reads used for pilon polishing), and it caused a premature stop codon at the 344^th^ exon position of the gene. The premature stop codon caused a loss of 157 base pairs (bp) and resulted in a truncated gene sequence of 354 bp. To rectify this issue, we compared the 354 bp sequence of the R2R3 Myb transcription factor gene, including the missing 157 bp sequence, with the corresponding gene sequence from the Barhee BC4 genome and corrected the sequencing errors. The alignment shows that the missing 157bp sequence is 100% similar to the R2R3 Myb transcription factor gene from the Barhee BC4. **a:** Comparison of the R2R3 Myb transcription factor gene sequence from PDK50 genome (354bp sequence length) and Barhee BC4 genome (507bp sequence length) . Indel mutations were marked in red color. **b:** Indel mutations details of R2R3 Myb transcription factor gene. **c:** Alignment of 354bp sequence of R2R3 Myb transcription factor gene against R2R3 Myb transcription factor gene from Barhee BC4. **d:** Alignment of the error corrected R2R3 Myb transcription factor gene sequence, including the missing 157bp sequence against the R2R3 Myb transcription factor gene from Barhee BC4 genome.

Supplementary Figure 11. Boxplot distributions analysis of fruit color phenotype (R/B) by genotypes of significant GWAS SNPs associated with fruit color in VIR sample groups. Samples were classified into two groups: (**a**) wild type group: samples with VIR^+^/VIR^+^ of VIR^+^/VIR^saf^ (n=25) and (**b**) VIRIM group: samples with VIR^IM^/VIR^IM^ genotype (n=61) and then performed the analysis of phenotype by genotypes of significant SNPs. Genotypes of SNP LG4s19036701 could not distinguish the fruit color in the VIRIM sample group, likely because of linkage on the same chromosome as the VIR gene, and so was excluded from this specific analysis. P-values were calculated using by **Wilcoxon statistical test.** The X-axis represents the SNP’s genotypes, and Y-axis represents R/B phenotypic value.

Supplementary Figure 12. Structural variation (deletion) present in candidate region of LG10s12886617 SNPs on LG 10. Clipped, discordant, unmapped, and indel reads from samples with LG10s12886617 SNP with homozygous allele alternative (ALT) to the reference genome was assembled and aligned against LG 10. The alignment of contigs 6 (12.7949 kb length) showed a 5 kb deletion in the region 12.8743Mb to 12.8794Mb of LG 10. This 5 kb deletion is located 800 bases from the pentatricopeptide repeat-containing protein gene and 7 kb away from the SNP LG10s12886617.

Supplementary Figure 13. Boxplot distributions analysis of fruits color phenotype by genotypes of significant GWAS SNPs in all 188 samples. The SNPs were identified using an unadjusted p-value 10e-7 as a cut-off value for determining the list of significant SNPs associated with the fruit's color phenotype (R/B).

Supplementary Figure 14. RNA seq analysis of expression of genes related to fruit ripening and pigmentation is present within the region of significant SNPs identified by an unadjusted p-value 10e-7 as a cut-off value. The gene expression analysis was performed across three or four replicates of fruit development stages of two fruit color varieties, Kheneizi (red fruit) and Khalas (yellow fruit). Each point of the X-axis is the development stage (post-pollination date) and the Y-axis is the mean of normalized expression read count across three or more replicates.

**Supplementary Table**

Supplementary Table 1**:** Genomic coordinates of  VIR *gene,* retrotransposon insertion (Ibn Majid), and locations of start lost codon and termination codon variant in LG 4 of PDK50 genomes.

| Allele TYPE | Features | Start | End |
| --- | --- | --- | --- |
| Retrotransposon Insertion  (VIRIM) | Exon1 | 65251488 | 65251623 |
|  | Exon2 | 65251695 | 65251824 |
|  | Exon3 | 65252695 | 65252935 |
|  | Ibn Majid (LTR retrotransposon) | 65252928 | 65264522 |
|  | Termination codon | 65252936 | 65252938 |
| Lost start codon (VIRsaf) | ATG/ATA SNP | 65251490 | 65251490 |
| Wild Type ( VIR+) | Exon 3 (CDS), 5’ of Ibn Majid | 65252695 | 65252927 |
|  | Termination codon (TGG/TGA) | 65264705 | 65264707 |

Supplementary Table 2. List of significant SNPs significantly associated with the (R/B) phenotype. An unadjusted p-value of 10e-7 was used as a cut-off value for identifying the list of significant SNP

| SNP ID | LG ID | SNP Position | P.value | MAF | FDR  P.value |
| --- | --- | --- | --- | --- | --- |
| LG4s65268794 | LG4 | 65268794 | 7.40E-12 | 0.416201117 | 2.62E-05 |
| LG10s12886617 | LG10 | 12886617 | 1.56E-10 | 0.19273743 | 0.000276496 |
| LG3s906369 | LG3 | 906369 | 3.29E-10 | 0.293296089 | 0.000388953 |
| LG4s19036701 | LG4 | 19036701 | 3.84E-09 | 0.17877095 | 0.003401362 |
| LG5s14425448 | LG5 | 14425448 | 4.98E-08 | 0.089385475 | 0.035277173 |
| MU008982.1s1030911 | MU008982.1 | 1030911 | 7.25E-08 | 0.203910615 | 0.042803883 |
| LG13s8766984 | LG13 | 8766984 | 2.58E-07 | 0.446927374 | 0.130612832 |
| LG5s4683788 | LG5 | 4683788 | 6.45E-07 | 0.170391061 | 0.26190628 |
| LG18s4603014 | LG18 | 4603014 | 6.66E-07 | 0.301675978 | 0.26190628 |
| LG13s912271 | LG13 | 912271 | 7.89E-07 | 0.11452514 | 0.279481236 |

Supplementary Table 3. List of genes detected around the potential region of SNPs from GWAS results associated with R/B phenotypes to have significant role in fruit ripening and pigmentation. SNPs were identified based on an unadjusted p-value cut-off of 10e-7

| Tag SNP ID | LG ID | Gene ID | Gene Description | Gene Distance from Tag SNP |
| --- | --- | --- | --- | --- |
| LG3s906369 | LG3 | PDK50.r1.LG3G00293750 | Protochlorophyllide reductase, chloroplastic | 3.5 kb |
| LG3s906369 | LG3 | PDK50.r1.LG3G00293700 | Basic helix-loop-helix (BHLH) DNA-binding superfamily | 83 kb |
| LG4s65268794 | LG4 | PDK50.r1.LG4G00377090 | R2R3-MYB transcription factor | 16 kb |
| LG5s4683788 | LG5 | PDK50.r1.LG5G00393890  PDK50.r1.LG5G00393990 | 4-coumarate:CoA ligase | 133 kb |
| LG5s4683788 | LG5 | PDK50.r1.LG5G00393910.1  PDK50.r1.LG5G00393950.1 | AP2/ERF and B3 domain-containing transcription factor family | 88 kb |
| LG5s4683788 | LG5 | PDK50.r1.LG5G00393980 | 4-coumarate:coA ligase 3 | 50 kb |
| LG5s4683788 | LG5 | PDK50.r1.LG5G00394020 | AP2/B3-like transcriptional factor family protein | 8 kb |
| LG5s4683788 | LG5 | PDK50.r1.LG5G00394040 | Myb family transcription factor family protein | 12 kb |
| LG5s4683788 | LG5 | PDK50.r1.LG5G00394080 | Chalcone-flavanone isomerase family protein | 87 kb |
| LG10s12886617 | LG10 | PDK50.r1.LG10G00073810 | Ethylene-responsive transcription factor 12 | 116 kb |
| LG10s12886617 | LG10 | PDK50.r1.LG10G00073870 | RING/U-box superfamily protein | 3 kb |
| LG10s12886617 | LG10 | PDK50.r1.LG10G00073880.1 | Myb family transcription factor family protein | 12 kb |
| LG13s912271 | LG13 | PDK50.r1.LG3G00148810 | AP2-like ethylene-responsive transcription factor | 36 kb |
